# The Role of Metabolic Changes in Shaping the Fate of Cancer-Associated Adipose Stem Cells

**DOI:** 10.3389/fcell.2020.00332

**Published:** 2020-05-15

**Authors:** Giulia Cantini, Alessandra Di Franco, Massimo Mannelli, Anthony Scimè, Mario Maggi, Michaela Luconi

**Affiliations:** ^1^Endocrinology Unit, Department of Experimental and Clinical Biomedical Sciences “Mario Serio,” University of Florence, Florence, Italy; ^2^Molecular, Cellular and Integrative Physiology, Faculty of Health, York University, Toronto, ON, Canada; ^3^Istituto Nazionale Biostrutture e Biosistemi, Rome, Italy; ^4^Azienda Ospedaliero Universitaria Careggi, Florence, Italy

**Keywords:** adipose precursors, metabolic reprogramming, Warburg effect, p107, adrenal tumors

## Abstract

Adipose tissue in physiological and in metabolically altered conditions (obesity, diabetes, metabolic syndrome) strictly interacts with the developing tumors both systemically and locally. In addition to the cancer-associated fibroblasts, adipose cells have also recently been described among the pivotal actors of the tumor microenvironment responsible for sustaining tumor development and progression. In particular, emerging evidence suggests that not only the mature adipocytes but also the adipose stem cells (ASCs) are able to establish a strict crosstalk with the tumour cells, thus resulting in a reciprocal reprogramming of both the tumor and adipose components. This review will focus on the metabolic changes induced by this interaction as a driver of fate determination occurring in cancer-associated ASCs (CA-ASCs) to support the tumor metabolic requirements. We will showcase the major role played by the metabolic changes occurring in the adipose tumor microenvironment that regulates ASC fate and consequently cancer progression. Our new results will also highlight the CA-ASC response *in vitro* by using a coculture system of primary ASCs grown with cancer cells originating from two different types of adrenal cancers [adrenocortical carcinoma (ACC) and pheochromocytoma]. In conclusion, the different factors involved in this crosstalk process will be analyzed and their effects on the adipocyte differentiation potential and functions of CA-ASCs will be discussed.

## Introduction: Obesity-Cancer Association

Obesity is a main risk factor for cancer development in many types of solid tumors, suggesting an association between these two pathologies and a new perspective of cancer as a metabolic pathology, which may open new therapeutic prospects. Though evidence reports obesity as a risk factor for the development of solid tumors (Kyrgiou et al., 2017), it is less clear whether excess body weight is associated with poorer survival in cancer patients. Some studies reported that being overweight or obese, defined by a body mass index (BMI) greater than 25 and 30 kg/m^2^, respectively, is associated with improved survival in some types of cancer (Lennon et al., 2016; Park et al., 2018). Regardless, cancer biology differs between obese and lean patients and this aspect must be taken into consideration when developing personalized therapeutic approaches.

**FIGURE 1:**
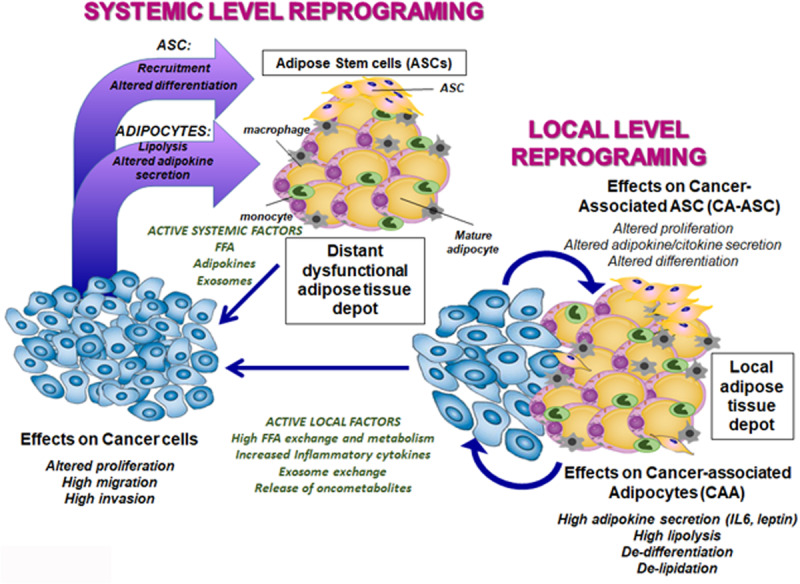
Cartoon of the systemic and local interaction occurring between adipose tissue depots and the developing solid tumor. Interaction between cancer and adipose tissue can occur at systemic levels through factors released by the tumor mass and affecting distant adipose depots both on ASC and mature adipocyte components. Moreover, altered adipose tissue as in obesity can reciprocally affect distant tumor mass by secretion of hormones and adipokines. In addition, a reciprocal interaction between cancer and adipose (ASCs and adipocytes) cells can occur locally on the adipose mass surrounding the developing tumor through exchange of different bioactive factors, which produce effects on cancer cells, adipose precursors and mature adipocytes, as described in the text.

Obesity can affect tumorigenesis and cancer progression at two different levels (Figure 1). First, a systemic one, as dysfunctional adipose tissue depots produce an altered profile of adipokines and cytokines affecting the distant tumor mass. Second, obesity also locally alters the adipose microenvironment of the tumor. In general, a crosstalk occurs between the local adipose tissue and the developing tumor mass to sustain tumor progression. In particular, studies focusing on breast cancer have demonstrated that adipose stem cell (ASC) populations have an altered differentiation potential whether derived from obese or lean subjects when cocultured with breast cancer cell lines. Moreover, these changes are associated with an enhanced ability to sustain breast cancer cell proliferation and *in vivo* tumorigenicity (Strong et al., 2013). These effects seem to be mainly mediated by the tumor-induced modulation of the leptin-estrogen axis in ASCs. An increase in leptin, estrogen receptor alpha (ERα) expression, and aromatase activity has been described in obese subjects in coculture with breast cancer, whose specific block reverted tumor progression and metastasis (Sabol et al., 2019).

## The Local Adipose Tissue Microenvironment of the Tumor

The tumor effect in reprogramming the adipose tissue microenvironment can act on differentiated cells (adipocytes), representing the bulk of cells in the fat depot, or on the fewer local ASCs (Figure 1). In addition, recruitment of mesenchymal cells at the tumor site from the bone marrow and other sources has been described (Kidd et al., 2012; Berger et al., 2016). Mobilization of ASCs into the peripheral blood has also been reported in obese cancer patients, suggesting an increased ASC recruitment and trafficking to the tumor site is associated with the obese condition (Bellows et al., 2011; Zhang et al., 2016). The majority of studies investigating cancer modulation of adipose precursors at tumor sites have been derived from breast cancers, due to the high content of fat in the mammary gland. However, local effect on adipose tissue has also been extensively studied in prostate, ovarian, adrenal, endometrial, and melanoma cancers.

The ability of the tumor to actively influence ASCs may depend on the direct cell–cell interaction, on the secretion of oncometabolites and signaling proteins, or on the active release of factors encapsulated in “cargoe-like” microvesicles and exosomes (Wu et al., 2019). The latter mechanism might be more effective in cell reprogramming, as these vesicles can contain different types of oncoproteins, miRs, mRNA, and metabolites, which might be selectively integrated in the target cells. Primary breast ASCs treated with exosomes derived from breast or ovarian cancer cells showed a tumor-associated myofibroblast phenotype. This was characterized by increased expression of α-SMA and production of tumor-promoting factors stromal-derived factor-1 (SDF-1), vascular endothelial growth factor (VEGF), CCL5, and transforming growth factor β (TGFβ), which promote the upregulation of the SMAD signaling (Cho et al., 2011, 2012; Song et al., 2017).

## The Adipose Stem Cell (ASC)

Stem cells play a pivotal role in maintaining the complex tissue architecture in physiology and in cell therapies due to their self-renewal capacity and the ability to differentiate into multiple cell lineages, providing potential therapeutic solutions for different diseases.

Among adult stem cells, the bone-marrow mesenchymal stem cells (BM-MSCs) (Chamberlain et al., 2007; Kokabu et al., 2016), are a multipotent cell type that can give rise not only to osteoblastic cells but also to other cell types, including adipocytes (Kokabu et al., 2016). Other BM-MSCs like adult stem cells can be isolated from almost every tissue including adipose (Mohamed-Ahmed et al., 2018) and interestingly from some tumors (Si et al., 2019). In the adipose tissue, this population represented by the ASCs are one of the most promising adult stem cell populations identified because they are ubiquitous and can be easily recovered and harvested in large quantities with no significant complication to donors (Si et al., 2019). The adipose tissue is a highly heterogeneous tissue for the co-existence of ASCs and different types of adipocytes (white, beige, brown), endothelial cells, pericytes, and immune competent cells. Moreover, differences in the properties and functions of the adipose tissue and its cell components have been identified, which are based on the fat depot location and the metabolic status of the subject.

Human ASCs, first isolated and characterized by Zuk et al. (2001), are not homogenous populations. To be considered ASCs they must display some specific features, such as plastic adherence, positivity for typical BM-MSC markers (CD73, CD90, CD105), and lack of expression of hematopoietic markers such as CD45, CD34, CD14, or CD11b, and of endothelial CD31 and Human Leukocyte Antigen DR (HLA-DR) surface molecules. Several reports show the ASCs can differentiate into multiple lineages both *in vitro* and *in vivo* (Cawthorn et al., 2012). ASC differentiation can be reproduced *in vitro* by selective induction with media containing lineage-specific inductive factors (Zuk et al., 2001, 2002). Although ASCs are of mesodermal origin, controversially they have been shown to differentiate into cells of ectodermal and endodermal origin as well (Zuk et al., 2001, 2002; Baglioni et al., 2009; Si et al., 2019).

ASCs can secrete several growth factors and cytokines critical for tissue repair and remodeling (Pinheiro et al., 2012; Yun et al., 2012) and for the improvement of vascularization and neo-vessel formation (Pinheiro et al., 2012). Moreover, it has been reported that ASC transplants may affect inflammatory processes by displaying immunosuppressive properties (Puissant et al., 2005; Fang et al., 2007; Crop et al., 2010). Unlike BM-MSCs, ASCs do not induce an allogenic lymphocyte response *in vitro*, and prevent lymphocyte proliferation induced by allogenic peripheral blood mononuclear cells or mitogens (Puissant et al., 2005). In two studies, the intravenous infusion of allogenic ASCs was effective in the treatment of severe acute graft-vs.-host disease, indicating that ASCs may be used for their immunomodulatory effects (Si et al., 2019). However, these ASC immunomodulatory properties might also play a role in contributing to the immunomimetism developed by some tumors to avoid any immune response that might be directed against them.

## Depot Differences for White Adipose Stem Cells

There are intrinsic differences in functional activity, secretion profile, and susceptibility to obesity-induced derangement in the adipose tissue depots according to the subcutaneous (SAT) or visceral (VAT) distribution. Importantly, while SAT expansion can be considered a protective response to the caloric load, excess fat deposition in VAT results in tissue dysfunction and correlates with the development of metabolic diseases. Differences in response to lipid excess are also present in ASCs of the two depots in humans (Baglioni et al., 2012) and mice (Lefevre et al., 2019). In humans, visceral ASCs (V-ASCs) display a reduced ability to differentiate toward functional adipocytes, which produce lower levels of the metabolic protective adipokine adiponectin, and are more prone to lipolysis compared to ASCs from subcutaneous adipose tissue (S-ASCs) obtained from the same subjects (Baglioni et al., 2012). Moreover, the differentiation potential and multipotency toward different cell types, such as chondrogenic, osteoblastogenic, neurogenic, and muscle, are significantly higher in S-ASCs than in V-ASCs (Baglioni et al., 2012), suggesting a strong intrinsic difference in precursor fate potential according to the depot origin. The metabolic pathways predominantly acting in S- and V-ASCs might underlie the differences observed in the precursor fate decisions (Lefevre et al., 2019). In general, the balance between cell proliferation and differentiation is controlled by glycolytic versus. mitochondrial respiration activity (Pattappa et al., 2011). Differentiation often requires a switch from a predominantly glycolytic metabolic program that sustains cell proliferation, characterizing an undifferentiated status of the cell, to an increase in mitochondrial oxidative phosphorylation (OXPHOS) (Wanet et al., 2015).

Visceral ASCs have a decreased adipogenic fate potential and an inability to differentiate toward the osteogenic lineage compared to S-ASCs. High-resolution nuclear magnetic resonance metabolomic comparisons of murine ASCs revealed that the shortcoming is associated with a more pronounced glycolytic metabolism, with reduced mitochondrial activity, resulting in an increased utilization of pyruvate to produce lactate in V-ASCs. A similar metabolic control exerted by glycolysis might be hypothesized to be acting in CA-ASCs where the crosstalk with the tumor cells results in a restriction of the differentiation potential toward adipocytes to support cancer progression.

Conversely, the tricarboxylic acid (TCA) cycle and mitochondrial respiration are more active in S-ASCs, resulting in a reduced production of lactate and an increased synthesis of citrate (Lefevre et al., 2019). Interestingly, citrate produced in the TCA cycle and transported in cytosol is necessary for fatty acid synthesis in mature adipocytes (Senyilmaz and Teleman, 2015). In addition to glucose, the other metabolite that fuels the TCA cycle is glutamine. Glutamine deprivation induces a Warburg-like effect and completely switches the S-ASC metabolism toward lactate synthesis (Lefevre et al., 2019).

## Adipose Precursor Metabolic Alterations Induced by the Tumor Microenvironment

Of note, when positing a potential cross talk between cancer cells and adipose tissue, the main target is represented by mature adipocytes that constitute around the 90% of the fat mass, while resident ASCs represent a limited component.

A shift of white adipocytes toward stem cell like properties has been described for numerous solid tumors (Cao, 2019). These cells are characterized by marked morphological changes such as de-lipidation and de-differentiation, free fatty acid release, and immunomodulatory adipokine secretion profile. In addition, the cells are distinguished by a metabolic shift, induced by the presence of cancer cells, from OXPHOS toward glycolysis, resulting in the so-called reverse-Warburg effect. In different types of cancer, this re-programming of the mature adipose cells is thought to occur to provide the cancer cells with the substrates to fuel β-oxidation and the oncometabolites necessary for growth and invasion (Dirat et al., 2011; Nieman et al., 2011; Romero et al., 2015; Zoico et al., 2018).

The initial concept developed by Otto Warburg was that tumor cells have a defect in the mitochondrial respiration that leads to the increased glycolysis observed in the tumor cells (Warburg, 1925, 1956; Weinhouse, 1956). However, the current “chicken-or-egg” question is if the Warburg effect is just a consequence of cancer due to a defective mitochondrial respiration or does it cause carcinogenesis. The reverse Warburg effect found in ASCs in response to the metabolic reprogramming induced by the tumor is an example of the occurrence of this shift without any mitochondrial defect (Poli et al., 2015; Armignacco et al., 2019). Indeed, the enhanced aerobic glycolysis occurring in the presence of highly active lactate dehydrogenase shifts pyruvate produced from glycolysis toward lactate and not to acyl-CoA to fuel TCA cycle. This induces a reduction in the rate of mitochondrial respiration even in the absence of functional defects of the organelle (Senyilmaz and Teleman, 2015; Devic, 2016).

The tumor interaction with the microenvironment can be described as a “parasitic” energy transfer from adipose cells, where catabolic processes (autophagy, glycolysis, lipolysis) produce energy-rich nutrients that are transferred to the tumor cells to fuel anabolic processes that sustain tumor growth and metastasis (Martinez-Outschoorn et al., 2011; Romero et al., 2015). In such a strict cycle, stromal catabolites (such as lactate, amino acids, and free fatty acids) promote tumor growth by acting as high-energy oncometabolites. Moreover, the tumor cells induce a metabolic shift in ASCs that triggers their fate to be more functional for the tumor growth.

To study the role of the mitochondria in tumor energy metabolism and in tumor-induced reprogramming, a mouse model known as tuning mitochondrial dysfunction (mTUNE) has recently been developed (Gaude et al., 2018). It is applied to investigate the reciprocal relationship occurring between mitochondrial alteration, glycolysis, and reductive carboxylation of glutamine in driving cell reprogramming and migratory activity (Valcarcel-Jimenez et al., 2017).

In addition to lactate secretion, as a consequence of pyruvate redirection during the Warburg effect in the tumor, acidification of the tumor microenvironment is also maintained. During late phases of development, hypoxic conditions occur when the tumor mass interferes with oxygen transport through loss of vascularization. The hypoxic-inducible enzyme, carbonic anhydrase that converts carbon dioxide and water into carbonic acid, protons, and bicarbonate, is upregulated, contributing to the maintenance of the acidic tumor microenvironment. Interestingly, carbonic anhydrase is involved in adipocyte differentiation (Weber, 2016). Thus, its modulation during acidification induced in the tumor microenvironment might be influencing cancer-associated adipocyte differentiation.

We have recently shown the occurrence of an *in vitro* shift in the fate of white adipose precursors, when primary cultures of human ASCs were induced to differentiate in coculture with the adrenocortical carcinoma (ACC) cell line, H295R (Armignacco et al., 2019). The number of mature adipocytes that were formed *in vitro* was severely attenuated and characterized by a reduction of intracellular lipid droplets. In addition, the cells expressed significantly lower levels of proteins associated with functional adipocytes, such as adiponectin, Fatty Acid-Binding Protein 4 (FABP4) and Hormone-Sensitive Lipase (HSL), and had reduced accumulation of intracellular lipid droplets. This shift was accompanied by an increased production of lactate and glucose uptake. Thus, a significant reprogramming of adipose differentiation in the presence of tumor cells was supported by a shift in the intracellular metabolism (Armignacco et al., 2019).

Of note, aggressive adrenocortical cancers are often characterized by hyper-activating mutation or upregulation of Wnt/beta catenin pathways (Assié et al., 2014) and hyper-methylation/repression of the G0/G1 switch gene 2 (G0S2) gene (Barreau et al., 2013; Mohan et al., 2019). Interestingly, both Wnt and G0S2 are physiologically implicated in the inhibition of adipogenesis, suggesting that common mechanisms between ACC tumor progression and ASC reprogramming might occur in the tumor microenvironment (Armignacco et al., 2019). Indeed, Wnt5 is upregulated in ASCs and its downregulation is necessary for adipogenesis (Christodoulides et al., 2009). In addition, G0S2 expression is involved in enhancing intracellular triglyceride accumulation and inhibition of lipolysis in adipocytes. This suggests that its repression, as found in the advanced and more aggressive forms of ACC (Mohan et al., 2019), may be associated with a reduced accumulation of lipid droplets not only in the tumor cells but also in the cancer-associated adipocytes. Further studies are needed to clarify if this association really occurs in advanced ACC and if it is limited to adrenocortical cancer.

## The Risk of Tumorigenicity of ASCs in Adipose Grafts Used for Regenerative Medicine

For many years, autologous adipose grafts used in medical procedures, such as lipofilling of healing wounds, scar remodeling, and after surgery removal of tumor masses, have been studied for their regenerative potential. Indeed, the presence of ASCs has been demonstrated to significantly improve the engraft and the reconstruction of the tissue architecture, due to the paracrine effects of ASC secretion and stimulation of adipocyte differentiation (Mashiko and Yoshimura, 2015). Interestingly, *in vitro* studies showed that the presence of scaffolding biomaterials introduced in the graft has the potential to stimulate ASC differentiation and enhancing adipose replenishment (Stellavato et al., 2017). Autologous fat graft procedures are of particular importance to reconstruction of resected tissue rich in adipose mass, such as breast cancer surgery. A recent meta-analysis excluded any increased risk of recurrence after fat graft reconstruction in breast cancer surgery (Agha et al., 2015). However, the safety of this approach in patients with cancer is currently debated due to the potential risk of ASCs to influence tumor recurrence (Tan and Loh, 2016; Mazur et al., 2018). Indeed, residual post-surgery cancer cells may influence ASCs present in the fat graft inducing proliferation and maintenance of a a stem-like state that may promote cancer restart. A predicament similar to what already has been demonstrated for the crosstalk occurring between the primary tumor mass and the ASCs present in the surrounding microenvironment for different solid tumors (Paino et al., 2017; Armignacco et al., 2019). Moreover, both resident and engrafted ASCs can stimulate angiogenesis (Paino et al., 2017). This may contribute to tumor recurrence by secretion of specific adipokines and factors, such as collagen and matrix components, that make the microenvironment more prone to tumor growth (Iyengar et al., 2003). The crosstalk between residual tumor and adipose microenvironment cells may be particularly important in driving the reciprocal fate of ASCs and tumor stem cells (Papaccio et al., 2017). Indeed, ASCs may contribute to the maintenance of a stem-like state and immunomodulatory properties of the cancer stem cells (Si et al., 2019), maintaining them quiescent and masked to the immune response for long time (Paino et al., 2017).

## The p107 Check Point in Controlling ASC Metabolism

Tumor-induced systemic wasting and cancer cachexia, characterized by a decrease in muscle mass and white fat accumulation, have been associated with increased thermogenic activity and browning of adipose tissue (Kir and Spiegelman, 2016). Factors produced by the tumor reprogram a shift in the adipose tissue inducing the “browning process.” Among these factors, the lipid mobilizing factor zinc-α2-glycoprotein (ZAG), highly expressed in breast, prostate, lung, and bladder tumors (Díez-Itza et al., 1993; Hale et al., 2001) has been demonstrated to promote lipolysis and inhibit lipogenesis in WAT (Taylor et al., 1992), while stimulating white adipose tissue browning and cachexia (Elattar et al., 2018). In a Lewis lung carcinoma mouse model of cancer cachexia, this reprogramming of white to brown differentiation fate is triggered locally and systemically by tumor-produced parathyroid-hormone-related protein (PTHrP), which upregulates the expression of those genes involved in thermogenesis in the adipose tissue (Kir et al., 2014). The causal effect is demonstrated by reversion of browning and muscle wasting cachectic effects through neutralization of PTHrP secretion (Kir et al., 2014) or genetic deletion of the PTH receptor (Kir et al., 2016). In parallel with an effect on “browning” of differentiated adipocytes there is an ASC effect of reduced adipocyte differentiation rate and increased differentiation into brite adipocytes. In some solid tumors that produce PTHrP, the tumor mass can reprogram adipose precursor fate not only by limiting adipogenesis, but also by shifting the differentiation fate toward thermogenic adipocytes. Both consequences appear to be more useful than having only white adipocytes to support tumor metabolic and proliferative requirements.

A member of the retinoblastoma susceptibility protein (Rb) gene family, Rbl1 (p107), might act as a CA-ASC metabolic checkpoint factor in this development. p107, a co-transcriptional repressor, is involved in cell cycle progression, as its over-expression is known to block cell cycle of many cancer cell lines and its loss associated with proliferative effects (Wirt and Sage, 2010). It has also been shown to determine ASC fate by regulating adipocyte differentiation through modulation of the glycolytic pathway. Adipocyte precursors depleted of p107 display an increased glycolytic flux associated with a reduction in the white adipocyte differentiation rate and a switch toward thermogenesis and “browning processes” (De Sousa et al., 2014; Porras et al., 2017). Importantly, lactate, the end-product of aerobic glycolysis, drives the commitment of unspecified adipocyte progenitors to brite fat *in vivo* (Porras et al., 2017). According to these findings, a suggestive role of p107 in driving CA-ASC fate following ASC interaction with tumor cells can be theorized. In this case, the cancer cell crosstalk with ASCs results in constraining white adipocyte differentiation potential and stimulating proliferation (Park et al., 2011; Koellensperger et al., 2014; Freese et al., 2015; Armignacco et al., 2019), similar to the process observed following p107 suppression in fat precursors (De Sousa et al., 2014). Intriguingly, coculture with ASCs reciprocally induces a reprogramming in the tumor cells, enhancing proliferation and invasion, as demonstrated for several solid tumors (Park et al., 2011; Cho et al., 2012; Klopp et al., 2012; Koellensperger et al., 2014; Armignacco et al., 2019; Xu et al., 2019).

Of note, p107 functions as a switch that regulates adipogenic fate into white versus. brown fat through repression of Peroxisome proliferator-activated receptor Gamma Coactivator 1-alpha (PGC-1α) and PR-DoMain containing 16 (PRDM16) genes (Scimè et al., 2005; De Sousa et al., 2014). In rodents, non-committed embryonic adipogenic progenitors have a metabolic state resembling aerobic glycolysis that is necessary for driving their pro-thermogenic fate. In the absence of p107, however, disruption of the glycolytic capacity reverts their differentiation fate from brown to white adipocytes, further confirming the role of p107 as a master switch controlling the adipose fate as well as the necessity of the aerobic glycolytic process to sustain the brown differentiation fate (Porras et al., 2017).

## A p107 Check Point in the Metabolic Shift of ASCs in Coculture With Adrenal Tumor Cells

We have recently developed a system of *in vitro* coculture between ASCs and two tumors of the adrenal affecting the steroid-secreting component of the gland, the ACC, and pheochromocytoma, which affects the catecholamine-secreting medullary adrenal part. One system makes use of ASCs derived from white adipose tissue (W-ASC) and a cancer cell line of ACC, H295R cells (Armignacco et al., 2019). The other is made up of cancer cells of pheochromocytoma (MPCs) and primary ASCs that exhibit brite cell characteristics (B-ASCs), since they are derived from the fat surrounding the pheochromocytoma lesion (unpublished results). Interestingly, we have previously demonstrated that this peri-adrenal visceral white fat has a great potential of generating brite adipocytes under the control of factors, which include catecholamines, produced at high levels by the pheochromocytoma mass (Di Franco et al., 2014).

**FIGURE 2:**
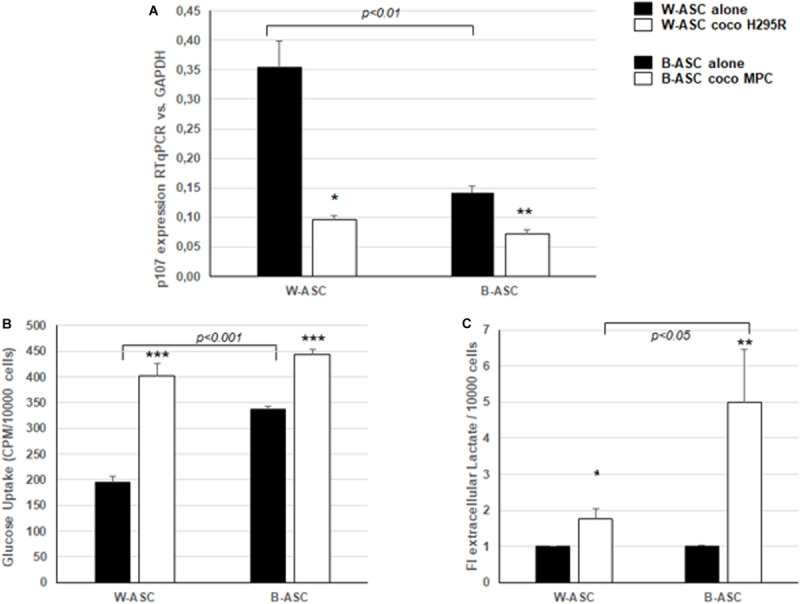
Differences in primary white ASC (W-ASC) and brite ASC (B-ASC) populations in monoculture (alone) or when cocultured (coco) with H295R cells or MPC cells, respectively. **(A)** Gene expression of p107 evaluated by quantitative RT-PCR and normalized for the house keeping gene GAPDH and related to a calibrator was expressed as mean ± SE of 2^– ΔΔ*CT*^ in *n* = 3 independent experiments. **(B)** Glucose uptake in W- and B-ASC expressed as mean ± SE of incorporated CPM/10^4^ cells after a 10 min pulse of cells with 2-deoxy-[^3^H]-D-glucose [1 μC/μL]; modified from Armignacco et al. (2019). **(C)** Extracellular lactate concentrations expressed as mean fold increase on cells alone normalized on 10^4^ cells ± SE of lactate measured in frozen conditioned media from ASCs cultured alone or in coculture for 7 days (colorimetric method, Siemens Healthcare, Tarrytown, NY, United States). Student’s *t*-test: *p* is indicated; and *p < 0.01, **p < 0.001; ***p < 0.0001 alone vs. coco.

p107 expression levels were significantly lower in B-ASCs compared with W-ASCs (Figure 2A), confirming that p107 might also interfere with the thermogenic program in human adipocyte precursors, and is not only restricted to mouse (Porras et al., 2017). Moreover, when in coculture with tumor cells, both types of ASCs displayed lower levels of p107, consistent with an altered differentiation potential. The decreased levels of p107 are also associated with a metabolic shift toward aerobic glycolysis. This is supported by the significant increase in ASC glucose uptake (Figure 2B), which was significantly higher in B-ASCs compared to ASCs in monoculture, suggesting a higher basal metabolism in the latter type of cells. Consistently, lactate production suggestive of aerobic glycolysis was increased in coculture in both systems, being significantly higher in B-ASCs (Figure 2C).

In white ASC cocultured with H295R cells for 7 days, Seahorse analysis^TM^ of basal glycolysis (Agilent Seahorse XF Glycolytic Rate Assay Kit #103344-100) showed a shift toward an increase of the aerobic glycolytic metabolic pathway (glycolytic proton efflux rate: cocultured cells vs. alone, 39,901 ± 1062 vs. 47,321 ± 958 pmol/min, *p* < 0.0001). We have already demonstrated that increased glycolysis is associated with a decreased differentiation potential and an increased maintenance of the stem potential and proliferation (Armignacco et al., 2019).

**FIGURE 3:**
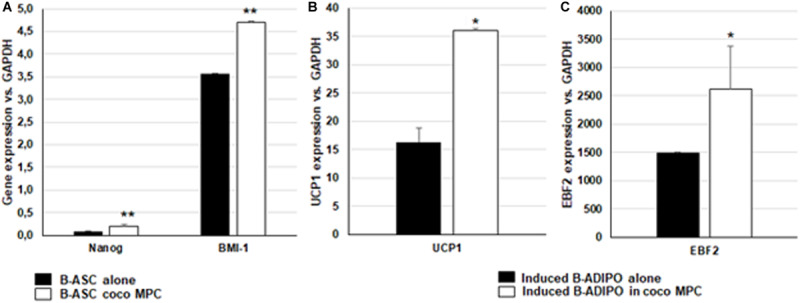
B-ASCs in coculture with MPC cells retain higher levels of stemness and higher levels of brite markers when adipogensis is induced *in vitro*. Gene expression of the stem genes Nanog and BMI-1 **(A)** or of brown markers UCP1 **(B)** and EBF2 **(C)** evaluated by quantitative RT-PCR, normalized for the house keeping gene GAPDH and related to a calibrator, was expressed as mean ± SE of 2^– ΔΔ*CT*^ in *n* = 3 independent experiments in B-ASC growth in monoculture or in coculture with MPC cells **(A)**, or when B-ASCs were induced to differentiate toward adipocytes with DIM cocktail in monocolture (ADIPO alone) or in the presence of MPC cells (ADIPO coco) **(B,C)**. Student’s *t*-test: *p < 0.01 and **p < 0.001 alone vs. coco.

**FIGURE 4:**
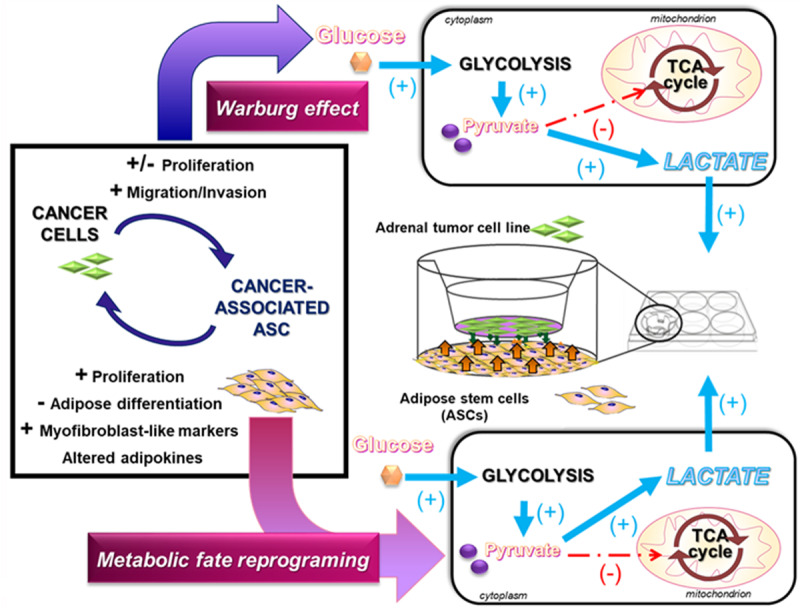
Metabolic shift affecting stem fate in ASCs in cocolture with adrenal tumor cells. Metabolic fate reprograming occurs between adrenal cancer cells and ASCs cocultured using ThinCert^TM^ tissue culture inserts for six-well plates with 0.4 μm pore size (Greiner Bio-One, Kremsmünster, Austria). In particular, an increased uptake of glucose is associated with a shift from mitochondrial OXPHOS to aerobic glycolysis, resulting in production of lactate and reduced utilization in the TCA cycle. This metabolic shift contributes to the reprograming in ASC fate (proliferation, differentiation, morphological changes, altered adipokine secretion, observed when they are grown in coculture with adrenal tumor cells).

Consistently, also for the B-ASCs in coculture with pheochromocytoma cells, an increased expression of genes associated with stemness (Nanog) and self-renewal (BMI-1) was evident (Figure 3A). In addition, when differentiation was induced *in vitro* in the presence of MPCs, B-ASCs the cocultured cells have an increased potential of differentiating toward brite adipocytes, expressing higher levels of Uncoupling Protein 1 (UCP1, Figure 3B) and of the transcription factor Early B-cell Factor 2 (EBF2, Figure 3C). These findings suggest how the metabolic pathway of aerobic glycolysis through potential regulation of p107 supports human cell undifferentiated conditions; upon differentiation a shift toward a pro-thermogenic program occurs. Figure 4 reports how the metabolic shift occurring in ASCs in coculture with adrenal tumor cells might affecting stem fate and functions.

## Depot Differences in ASC Response to Tumor Crosstalk

Metabolic circumstances, in particular obesity, can influence ASC characters, shaping the adipose precursors toward a cancer-sustaining phenotype. Differences in ASC response and interaction with the tumor mass, according to the metabolic profile of the patient, are supported by the higher extent of recruitment of circulating ASCs to the tumor mass in obese versus. lean humans and mice (Cozzo et al., 2017). Moreover, V-ASCs in obese males are shown to contribute to prostate cancer progression (Zhang et al., 2016).

**FIGURE 5:**
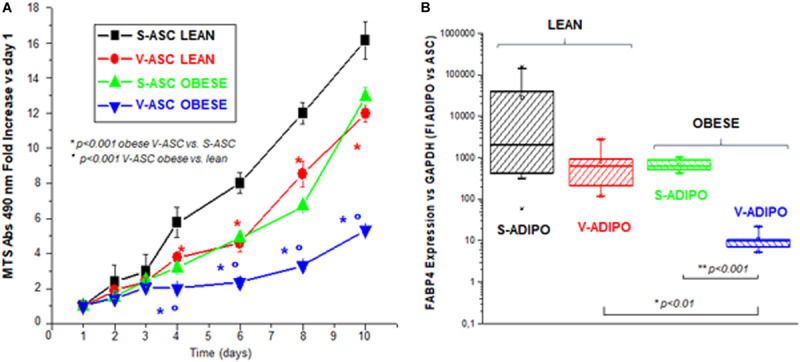
Differences in ASCs according to their fat origin. Primary ASC populations were obtained from subcutaneous (S-) or visceral (V-) adipose tissue (AT) of *n* = 4 and *n* = 5 independent lean or obese subjects, respectively. **(A)** Cell proliferation was assessed by MTS assay for the indicated time intervals in plated cells grown in parallel and expressed as mean fold increase ± SE of MTS absorbance vs. day 1. **(B)** Box charts of FABP4 gene expression, normalized for the housekeeping gene GAPDH and related to a calibrator, assessed as a marker of differentiation by quantitative real time RT-PCR in adipocytes (ADIPO) obtained *in vitro* after the parallel induction with DIM adipogenic medium (Baglioni et al., 2012) of ASC derived from SAT and VAT of *n* = 4 and *n* = 5 independent lean or obese subjects. Student’s *t*-test: *p*-values are indicated.

The ASCs derived from abdominal VAT compared to SAT of obese versus. lean subjects display a decreased proliferation rate (Figure 5A). Additionally, they have a diminished ability to differentiate into functional mature adipocytes, as shown by a decreased gene expression of FBP4 gene, a marker of mature terminally differentiated adipocytes (Figure 5B). Consistently with the hypothesis that V-ASCs rather than S-ASC are more susceptible to be reprogrammed by cancer crosstalk, the majority of solid tumors develop in visceral organs in close contact with visceral fat pads, with the exception of tumors of the skin, such as melanoma. V-ASCs rather than S-ASCs have been described to be more prone to sustain endometrial cancer survival and progression of endometrial cancer in a mouse xenograft model (Klopp et al., 2012). Thus, suggesting that ASC origin can drive different responsiveness in tumor induced cell reprogramming.

## Conclusion

The findings reported in this review reveal that the elevated plasticity of the adipose tissue is not confined to the mature adipocytes but is present and active in the ASCs in different pathophysiological contexts, including obesity and cancer. Such plasticity in reprogramming ASC fate and functions is strictly regulated by the intracellular metabolic equilibrium through the action of different factors. The regulatory pathways and the factors involved therein might represent valuable druggable targets for the development of novel therapeutic approaches to pathologies such as obesity and cancer.

## Data Availability Statement

The raw data supporting the conclusions of this article will be made available by the authors, without undue reservation, to any qualified researcher.

## Author Contributions

GC, AS, MMN, and ML contributed to conception and design of the experimental part of the study. GC, AS, MMG, and ML contributed to conception and design of the review structure and interpretation of the literature. GC and AD performed the experiments shown. ML wrote the first draft of the manuscript. GC, AS, and MMG wrote sections of the manuscript. All authors contributed to manuscript revision and read and approved the submitted version.

## Conflict of Interest

The authors declare that the research was conducted in the absence of any commercial or financial relationships that could be construed as a potential conflict of interest.
